# *Camponotusfloridanus* Ants Incur a Trade-Off between Phenotypic Development and Pathogen Susceptibility from Their Mutualistic Endosymbiont *Blochmannia*

**DOI:** 10.3390/insects9020058

**Published:** 2018-06-01

**Authors:** Veronica M. Sinotte, Samantha N. Freedman, Line V. Ugelvig, Marc A. Seid

**Affiliations:** 1 Department of Biology, Program of Neuroscience, University of Scranton, Loyola Science Center, Scranton, PA 1851-4699, USA; samantha-freedman@uiowa.edu (S.N.F.); marc.seid@scranton.edu (M.A.S.); 2 Centre for Social Evolution, Section for Ecology and Evolution, Department of Biology, University of Copenhagen, Universitetsparken 15, DK-2100 Copenhagen, Denmark; 3 Department of Pathology, University of Iowa, 1080 Medical Laboratories, 500 Newton Road, Iowa City, IA 52242-8205, USA

**Keywords:** *Camponotus*, primary endosymbiont, melanisation, caste polymorphism, disease susceptibility, *Metarhizium*

## Abstract

Various insects engage in microbial mutualisms in which the reciprocal benefits exceed the costs. Ants of the genus *Camponotus* benefit from nutrient supplementation by their mutualistic endosymbiotic bacteria, *Blochmannia*, but suffer a cost in tolerating and regulating the symbiont. This cost suggests that the ants face secondary consequences such as susceptibility to pathogenic infection and transmission. In order to elucidate the symbiont’s effects on development and disease defence, *Blochmannia floridanus* was reduced in colonies of *Camponotus floridanus* using antibiotics. Colonies with reduced symbiont levels exhibited workers of smaller body size, smaller colony size, and a lower major-to-minor worker caste ratio, indicating the symbiont’s crucial role in development. Moreover, these ants had decreased cuticular melanisation, yet higher resistance to the entomopathogen *Metarhizium brunneum*, suggesting that the symbiont reduces the ants’ ability to fight infection, despite the availability of melanin to aid in mounting an immune response. While the benefits of improved growth and development likely drive the mutualism, the symbiont imposes a critical trade-off. The ants’ increased susceptibility to infection exacerbates the danger of pathogen transmission, a significant risk given ants’ social lifestyle. Thus, the results warrant research into potential adaptations of the ants and pathogens that remedy and exploit the described disease vulnerability.

## 1. Introduction

Insects from the orders Coleptera, Blattodea, Pscopotera, Phithiraptera, Diptera, Hemiptera, and Hymenoptera engage in obligate mutualisms with primary bacterial endosymbionts, in which the nutritional benefits received by the host outweigh any costs of accommodating its partner [1]. Such mutualisms are characterized by long coevolutionary relationships, strict vertical transmission, high partner fidelity, symbiont genome reduction, and intracellular isolation of the symbiont in specialized cells (bacteriocytes) and tissues (bacteriomes) [1,2,3]. The endosymbionts’ nutritional supplementation promotes host fitness, ultimately resulting in ecological expansion and evolutionary diversification of the symbiotic partners [4]. Due to their aligned fitness interests, the host accommodates the symbiont via complementary metabolic support [5,6,7] and modification of its immune responses [4]. Pea aphids exhibit dramatically reduced bacterial immune defenses compared to other insects, which may be due, in part, to coevolution with their primary endosymbiont as well as specific life-history traits [8,9]. These reduced defenses allow the host to tolerate their primary endosymbiont, *Buchnera aphidicola*, but in turn can facilitate the colonization of pathogenic bacteria [10,11]. Unlike the pea aphid, *Sitophilus* weevils only demonstrate local downregulation of their immune response within the bacteriome [12,13], yet upon bacterial infection, *Sitophilus oryzae* symbiotic larvae raise a weaker systemic immune response than aposymbiotic larvae, suggesting that the weevils too may suffer increased susceptibility to pathogens [13]. While downregulating immune responses to tolerate the symbionts, these insects also regulate their symbiont through antimicrobial peptides, lysozyme production, and/or cell death pathways [5,14,15,16,17]. Thus, insect hosts likely incur a cost in tolerating and regulating their symbiont, which may result in reduced survivorship when challenged with pathogens or parasites.

Ants of the genus *Camponotus* host the primary endosymbiont *Blochmannia*, which supplements key nutrients, and they retain immune modifications to accommodate the symbiont. Similar to other primary endosymbionts, *Blochmannia* have extensive species-specific coevolutionary history with their host, vertical transmission, and residence within bacteriocytes intercalated in the ants’ midgut tissue [18,19,20,21,22]. The endosymbiont upgrades the host diet through recycling nitrogen and aiding in the synthesis of amino acids, fatty acids, and nucleotides [23,24,25,26], which are key in their pupal stage given the symbiont’s proliferation [27,28], gene expression [29,30], and the high fecundity of colonies compared to those with experimentally-reduced *Blochmannia* [25,29,31]. The ants tolerate the symbiont within bacteriocytes and ovaries; for example, *Camponotus floridanus* ants alter their expression of immunomodulators to reduce their immune response within these tissues [32,33]. The rest of the body maintains normal immune gene expression [32] unless *Blochmannia floridanus* is found in the haemocoel, at which point an immune response is mounted [34] that indicates the hosts’ readiness to prevent the spread of symbionts. However, it is unclear whether *B. floridanus* primes the host immune system, thereby promoting general pathogen resistance, or if the tolerance and regulation it induces in its host exacerbate pathogen susceptibility. Therefore, *C. floridanus* reaps crucial benefits from *B. floridanus’* nutrient supplementation, but the consequences of immune modification remain ambiguous.

Unlike other insects that host primary endosymbionts, *Camponotus* ants live in colonies and have a highly social lifestyle, offering a unique opportunity to study how the costs and benefits apply in a social setting. Classical theory predicts that colony size and worker polymorphism enhance colony efficiency and fitness [35,36]. Nutrient supplementation by *Blochmannia* directly benefits the colonies’ fecundity (e.g., colony size) [25,29,31]; however, *Blochmannia*’s role in affecting physical variation in worker castes, a trait hypothesized to contribute to *Camponotus*’ success as one of the most prevalent ant genera [37], has not been investigated. Conversely, if *Camponotus* suffer increased pathogen susceptibility due to their symbiont, the high density, relatedness, and contact rates in ant colonies will dramatically increase the risk of pathogen contraction, transmission, and outbreak of epidemics [38,39], which is a severe cost that must be offset by individual and/or social immune defenses. Consequently, *Camponotus*’ social lifestyle may exacerbate the costs and benefits it sustains from the symbiosis.

Here, we investigate the potential trade-off between nutritional support and altered immune defense that *Camponotus* ants might incur from hosting *Blochmannia*. We utilized *C. floridanus* colonies with their associated *B. floridanus* symbionts and created experimental colonies with ordinary and reduced symbiont levels. We then evaluated secondary benefits of the symbiont nutrient supplementation on individual growth and colony-level worker polymorphism. Furthermore, we examined altered immune defenses by quantifying cuticular melanisation, which is an important first barrier against parasites and pathogens [40,41]. Lastly, we measured ants’ susceptibility to the generalist entomopathogenic fungus *Metarhizium brunneum*, which infects its host by penetrating the cuticle and proliferating within the body [42] and can cause epizootics within colonies if not mitigated by the ants [38,39]. Our findings provide preliminary evidence that the symbiont supports increased body size and worker polymorphism. Our data further suggests that while *B. floridanus* promotes cuticular melanisation, ants with the symbiont exhibit reduced pathogen resistance. The underlying mechanisms of these observations remain unknown but warrant further investigation into this intriguing mutualism.

## 2. Materials and Methods

### 2.1. Ant Culture and Symbiont Reduction

Queen-right colonies of *Camponotus floridanus* were collected in Gainesville, Florida, in October 2014. Colonies were kept in plastic containers, each with an opaque humidified nesting chamber, at a constant temperature (22.8 °C) on a 12-h/12-h day/night cycle. Ants received a diet of 1 M sucrose solution and freshly killed *Tenebrio molitor* larvae three days per week for one year to allow the colonies to establish. 

**Figure 1:**
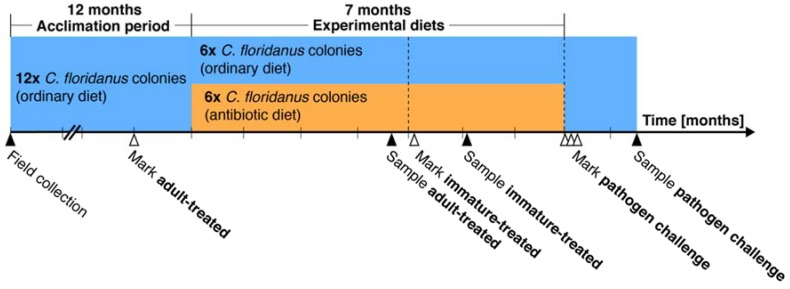
Timeline of the study, including onset and end of the experimental diet treatments and collection of ants for experimental assays. Colonies were provided with an ordinary diet (blue) for one year to acclimate to laboratory conditions and then fed an ordinary or antibiotic (orange) diet for seven months. Marking (white arrows) of adult ants prior to, and newly eclosed ants four months into the experimental diet treatment enabled creation of two colour-coded developmental groups: ants only receiving the experimental diet as adults (adult-treated) and ants receiving the experimental diets through the immature stages (from egg or larvae; immature-treated). Ants from these two age-controlled developmental treatment groups were sampled (black arrows) for head width, melanisation, and/or survivorship assays. Dashed lines indicate when the colonies were censused.

A total of twelve *C. floridanus* colonies of similar size were used, whereof some were provided with an ordinary (n = 6) and others an antibiotic (n = 6) diet for seven months (illustrated in Figure 1). Following this period, all colonies received the ordinary diet, comprising 1 M sucrose solution and *T. molitor* larvae. The antibiotic diet consisted of a two-week diet cycle, where in the first week, ants received a diet consisting of a 1 M sucrose with 1% (*w*/*v*) rifampicin (Sigma-Aldrich, Saint Louis, MO, USA) solution and *T. molitor* larvae three days per week, and in the second week, ants received the antibiotic-devoid diet on one of the three days. A rifampicin diet was selected as it has been widely used within *Camponotus* species to reduce or eliminate *Blochmannia* [21,25,29,31,43], as indicated by FISH microscopy and qRT-PCR [25,31]. Thus, we considered *B. floridanus* to be reduced from the fourth month onward in colonies that received the antibiotic diet. Further, rifampicin causes no visible damage to midgut tissues [21] or physiological changes attributed to toxicity [29,31], and no gut microbiota other than *B. floridanus* has been identified in *C. floridanus* that may be affected by the antibiotic diet [25]. Finally, preliminary experiments on *C. floridanus* colonies indicated that the antibiotic diet had a significant effect on cuticular colour (Supplementary Materials, Methods, and Results 1; Figure S1). 

### 2.2. Aging of Ants

All ants in the experiment were aged, given that age can affect cuticular melanisation [44], and age-dependent melanisation was confirmed in *C. floridanus*, with cuticular melanisation complete at approximately 30 days post-eclosion (Supplementary Materials Methods and Results 2; Figure S3). Ants were marked on the abdomen with enamel paint, with newly eclosing ants marked using different colours, resulting in ants colour-coded for age.

Two developmental groups were identified, as indicated in Figure 1, in order to assess the effects of *B. floridanus* at different stages of development. The first group of ants was termed “adult-treated”, because they were marked one month prior to the seven-month period of experimental diets, and thus were at least 30 day-old adults when the period began. They served as a control for the effects that the respective diets might have on fully developed ants. The adult-treated ants were sampled after receiving the experimental diets for four months. The other group is referred to as “immature-treated”. These ants were marked upon eclosion four months into the experimental diet period; that is, in colonies on the antibiotic diet, ants developed through the immature stages (egg or larvae) to adults with reduced *B. floridanus* levels. The immature-treated workers were sampled 30 days after marking and were used to assess the consequences of developing with reduced *B. floridanus* levels.

### 2.3. Head Width, Melanisation, and Worker Polymorphism Assays

Three colonies on the antibiotic diet and three on the ordinary diet were selected to assay the head width and melanisation of minor workers in the two developmental groups; three to six ants per colony were assessed for each combination of diet and age group. Ants were positioned with the dorsal side of their thorax visible to assess cuticular colour (immature-treated: n_antibiotic_ = 18, n_ordinary_ = 12; adult-treated: n_antibiotic_ = 9, n_ordinary_ = 9) and it was ensured that their head and thorax were flattened against the surface to measure head width (immature-treated: n_antibiotic_ = 9, n_ordinary_ = 12; adult-treated: n_antibiotic_ = 9, n_ordinary_ = 9). Ants were then photographed with a digital camera under normalized light and magnification next to a standardized grey scale and ruler. Head width and melanisation were measured using ImageJ 1.37 software. Head width was measured as the distance between the region behind both eyes, which is known to correlate with scape length and is a common proxy for body size [45]. The relative brightness of the cuticle was used as a measure of melanisation, with darker coloration corresponding to higher levels of melanisation. Photographs were converted to 16-bit greyscale images and the mean grey value within a pixel range of 0 (darkest) to 255 (brightest) was determined from a representative area of 1500 to 1600 pixels (45 pixels/mm) on the dorsal region of the thorax. 

To assess worker polymorphism, a census of major and minor workers was completed across colonies after seven months on the respective diets, with one of the antibiotic-diet colonies exempt due to an ant escape during the experimental period. The overall number of workers removed for the assessment of head width and melanisation were added to the total number of minors in the census. In addition, a census was obtained four months into the experimental diet period to demonstrate how colony size changed due to the different diets (Supplementary Materials Methods and Results 3; Table S2).

### 2.4. Fungal Entomopathogen 

An isolate of the fungal entomopathogen *Metarhizium brunneum* was obtained from the American Type Culture Collection (batch 93-09, media 325, ATCC#90448) and cultured as described in [46]. Conidiospores were harvested from Sabouraud dextrose agar (SDA) plates and suspended in 0.05% Triton-X solution. Viability was assessed to 91% germination on SDA plates, and a suspension of 3.3 × 10^6^ conidiospores/mL was created.

Prior to challenging the ants with *M. brunneum*, the antibiotic diet ceased for 40 days to reduce potential stress caused by the antibiotic itself [31] without increasing *Blochmannia* levels [21]. Minor workers 30 to 40 days-old post-eclosion from three colonies on the antibiotic diet and three on the ordinary diet were used; that is, they received the respective diet treatments as brood, but the ordinary diet as adults. A sham solution of 0.7 μL sterile 0.05% Triton-X (n_antibiotic_ = 12, n_ordinary_ = 12) or 0.7 μL of the conidiospore suspension (n_antibitoic_ = 15, n_ordinary_ = 15) was topically applied to the dorsal side of each ant’s thorax, and then ants were individually placed in Petri dishes (Ø = 5 cm) with moistened sterile cotton wool. The antibiotic diet reduced the number of ants the colonies produced, as observed in other studies [25,30,31], and thus limited the number of age-controlled ants available for the pathogen challenge. The ants were monitored for 10 days and their mortality recorded. On the day of death, cadavers were surface-sterilized by serial immersion in 70% ethanol, 8% sodium hypochlorite, and sterile water, and then left in humid conditions for 14 days to track fungal sporulation from the cadavers. 

### 2.5. Statistical Analysis

All statistical analyses were carried out in R version 3.3.2 [47], using the R-Studio editor version 1.0.153. General linear mixed models (LMM) were used to determine the fixed effects of *diet* and *development group* on head width and melanisation, specifying *colony* as a random effect. The lmer function and packages lme4 and lmerTest were used to create the models, and backwards model reduction was conducted to obtain the minimum adequate model. Post hoc pairwise comparisons of *diet* and *development group* were conducted using least-squared means with Tukey *p*-value adjustment, employing the lsmeans package. A generalized linear model with a binomial response and logit link was used to determine the fixed effect of *diet* and *colony size* on the number of major relative to minor workers, with *colony* as a random effect. The model used the glmer function and underwent backward model reduction. Collinearity among the fixed effect *diet* and *colony* size was tested using a Wilcoxon rank sum test with *diet* as grouping factor. Survival analysis was performed using a Cox proportional hazard regression model and likelihood ratio test statistics, with separate models created to test the effect of *diet* on baseline mortality (in sham-treated individuals) and the effect of *diet* on pathogen-induced mortality (in fungus-treated individuals). *Treatment* was included as a fixed effect and *colony* as a random effect. 

## 3. Results

**Figure 2:**
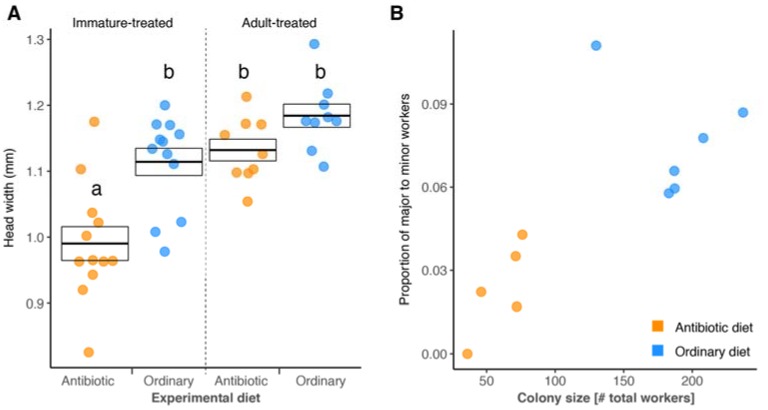
Reduced *B. floridanus* levels affect *C. floridanus’* development by decreasing body size and relative worker polymorphism within colonies. (**A**) Head widths of minor worker ants receiving antibiotic (orange dots) or ordinary diet (blue dots) during (immature-treated) or after (adult-treated) development from egg to adult. Overlaying boxes indicate the mean ± SE; letters specify significant post hoc groups (*α* = 0.05). (**B**) Proportion of major-to-minor workers and colony size after seven months on the antibiotic (orange dots) or ordinary diet (blue dots).

Head width, and hence growth, of minor workers was significantly affected by an interaction of diet and developmental group (Figure 2A; LMM; overall likelihood ratio test (LR) χ^2^ = 8.273, *df* = 1, *p* = 0.004). Ants developing from egg to adult on the antibiotic diet thus had significantly smaller head widths than those developing on the ordinary diet and those receiving the experimental diets as adults (post hoc comparison, all *p* < 0.001). This effect was further confirmed in callow workers (Supplementary Materials Methods and Results 2; Figure S2A). No significant difference was found in the head width of ants for the remaining combinations of diet and developmental group (Figure 2A; post hoc comparisons, all *p* > 0.05). Upon plotting the proportion of major-to-minor workers against colony size and diet, it became clear that the two fixed effects were confounded (Figure 2B; Wilcoxon rank sum; *W* = 0, *p* < 0.006). Diet was thus removed from the model, leaving colony size to have a significant, positive effect on the proportion of major-to-minor workers (LR χ^2^ = 6.507, *df* = 1, *p* = 0.011).

**Figure 3:**
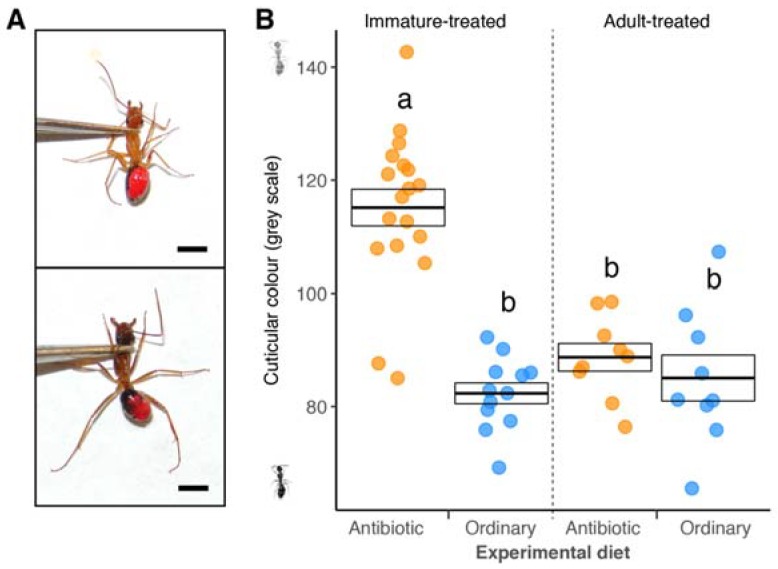
Cuticle melanisation decreases in *C. floridanus* ants that have reduced *B. floridanus* levels during development. (**A**) Example of standardized photos used to measure the cuticular colour and head width (greyscale values of 110.0 and 80.8 for the upper and lower frame, respectively); the scale bar is equivalent to 2 mm. (**B**) Cuticular colour (displayed as a greyscale) of minor worker ants receiving an antibiotic (orange dots) or ordinary diet (blue dots) during or after development (immature- and adult-treated, respectively). Overlaying black boxes indicate the mean ± SE; letters specify significant post hoc groups (*α* = 0.05).

The cuticular colour of ants was significantly affected by an interaction between diet and developmental group (Figure 3; LMM; overall χ^2^ = 18.065, *df* = 1, *p* < 0.001). Ants on the antibiotic diet during development exhibited a lighter cuticle (Figure 3A), which was significantly different from those on the ordinary diet (Figure 3B; post hoc comparison, *p* < 0.002) and also to ants that received the ordinary and antibiotic diets as adults (post hoc comparison, *p* = 0.003 and *p* < 0.001, respectively). The cuticle colour of ants from the remaining combinations of diet and development group did not differ (Figure 3B; post hoc comparisons, all *p* > 0.05). This effect was not observed in callow workers (Supplemental Materials, Methods, and Results 2; Figure S2B).

**Figure 4:**
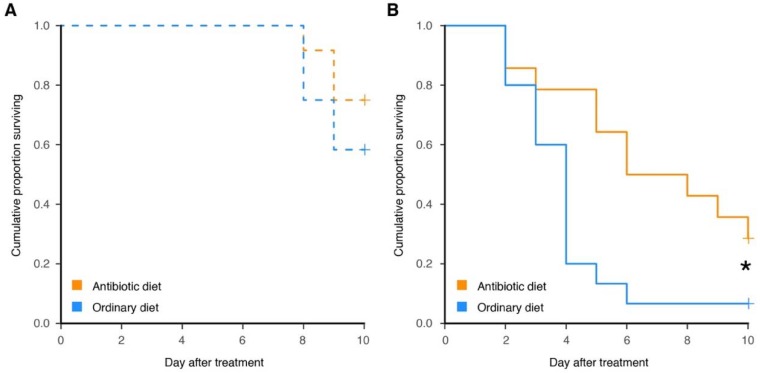
*C. floridanus* ants with reduced levels of the symbiont *B. floridanus* during development (**A**) exhibit no difference in mortality after sham-treatment (*p* = 0.360) and (**B**) higher survival after exposure to the fungal pathogen *M. brunneum* (*p* = 0.012), with the significant difference indicated by the asterisk. Censored data points are displayed as crosses.

Ants that developed on the antibiotic diet or ordinary diet exhibited similar mortality to the sham-treatment group (Figure 4A; Cox regression: overall LR χ^2^ = 0.830, *df* = 1, *p* = 0.360), whereas ants that received the antibiotic diet had a significantly lower mortality when challenged with *M. brunneum* (Figure 4B; Cox regression: overall: LR χ^2^ = 6.47, *df* = 1, *p =* 0.012). Ants with normal symbiont levels were 3.02 times more likely to die from fungal challenge than ants with reduced symbiont levels, and the disease progressed faster (reaching 80% on day 4 after treatment). Thus, despite a limited sample size, the experiment had sufficient statistical power to detect differences between groups. Sporulation characteristic of *M. brunneum* was observed in all but one cadaver that received the fungal treatment, which was excluded from the analysis. No sporulation was observed from cadavers that received the sham treatment.

## 4. Discussion

Reduction of the primary endosymbiont *B. floridanus* negatively influenced growth and development of its host *C. floridanus*, yet increased the ants’ resistance to a fungal pathogen. The direct benefits of the symbionts’ nutrient provisioning and costs to the host immune response have clear secondary consequences at the colony level. 

The smaller body size of minor workers as well as the reduced colony size and major-to-minor worker ratio in symbiont-reduced colonies demonstrates *B. floridanus*’ key nutritional support for *C. floridanus*’ individual and colony development. Body size can be influenced by an interplay of internal and external factors such as (epi-)genetics, social interaction, nutrition, competition, and physical environment [48]. The symbiont’s positive effect on body size indicates that the nitrogen and amino acids it supplies to larvae and nutrient-deprived pupae [25,29,30] contribute to a carbon and nitrogen balance crucial to brood development [49,50] and are potentially influential in worker caste determination [51,52,53]. In *C. floridanus*, epidermal growth factor receptor (Egfr) regulates worker size variation, with dramatic increases in expression in late larval instars of minor workers that restrain their size, as determined by global and local DNA methylation levels [45]. Recent studies suggest that bacterial symbionts may play a role in their host’s DNA methylation [54,55,56], and the shift in size observed in our study implies that *B. floridanus* might influence the Egfr pathway and thus development of minor workers. The smaller size of symbiont-reduced colonies, similar to in other studies [43], and corresponding reduced major-to-minor worker ratio indicates that *B. floridanus* may contribute to worker polymorphism directly through supporting individual body size and indirectly by promoting colony size. To disentangle the relative contributions of direct and indirect effects of defaunation on major-to-minor worker ratios in *C. floridanus* colonies, it would be necessary to obtain worker ratios from small colonies on ordinary diets. Still, the symbiont-supported development may facilitate caste-dependent division of labour in defense, resource retrieval, food processing, and brood care [36], which often improves the colony efficiency [35,48] and may in part explain the success of *Camponotus* as a genus [37]. 

The lighter, less melanised cuticle in ants with reduced *B. floridanus* levels implies that the symbiont is key to *C. floridanus*’ cuticle development, further emphasizing the importance of nutrient supplementation. *Blochmannia*, similar to the primary endosymbiont of *Cardiocondyla obscurior* ants, retain tyrosine biosynthesis pathways [6] that are highly expressed during the pupal stage and early adulthood, which coincide with the time point where the symbiont is most abundant [27,28,29,30,57]. Tyrosine is the molecular precursor to DOPA and dopamine, which can be oxidized to melanin, contributing to the melanisation (darkening), or acylated to *N*-acetyldopamine (NADA) and *N-β*-alanyldopamine (NBAD), facilitating sclerotisation (hardening) of the cuticle [58], both which occur in the pupal and callow stages in *C. floridanus*. Thus, the lighter cuticle of ants with reduced *B. floridanus* may indicate that the tyrosine synthesized by the symbiont is vital for melanisation. Supplementation of tyrosine or tyrosine marked with heavy isotopes to the antibiotic diet would allow a first test of whether cuticular melanisation can be rescued. Considering the general role of the tyrosine pathway in melanisation [59], a surprising contradictory result has been found in *Camponotus fellah*, where antibiotic-treated workers had higher cuticle melanisation and encapsulation rates than control workers [43]. Unfortunately, these studies did not control for worker age, although older workers can exhibit darker cuticular colour [44] and differential physiological immune responses [44,60,61,62]. Similar to the present study, de Souza and colleagues found that colony census decreased after four months of antibiotic treatment [43], implying that colonies comprised elevated old-to-young worker ratios. Measuring phenotypic traits of randomly selected workers at this or later time points of ongoing antibiotic treatment might thus confound the effects of diet treatment and age. Moreover, the antibiotic treatment itself may interfere with immune function, as evidenced by divergent relationships of encapsulation rate and endosymbiont levels in treated and control *C. fellah* workers [43]. We attempted to limit this effect by only assessing pathogen resistance 40 days after the end of the antibiotic diet. Besides these differences in experimental design, the divergent results may also be explained by differential regulation of pathways that convert dopamine to melanin, NADA, and NBAD, leading to different cuticle colouration [58,63,64,65]. Thus, *C. fellah* may utilize tyrosine differently when *Blochmannia* is reduced, which may be attributed to the species-specific differences in habitat and geographic range, with *C. floridanus* living in the subtropics of the New World and *C. fellah* living in arid climates of the Old World [66,67,68]. Further study of *Blochmannia*’s effect of the described molecular pathways and cuticular sclerotisation may clarify this discrepancy. Thus, differences in experimental design or the ant’s regulation of melanisation and sclerotisation likely explain the striking discrepancy of results.

Our data thus suggests that *B. floridanus* plays a crucial role in enhancing the integrity of the cuticle, which serves as a protective outer barrier and first line of defense [41]. The cuticle is of particular importance in protection against entomopathogenic fungi that infect their host by penetrating the host cuticle [42]. Moreover, the degree of cuticular melanisation has in a number of insects been linked to increased pathogen resistance [59,69], although melanin used in cuticle formation and internal immune responses (wound healing or encapsulation) appears to be regulated by different pathways [70]. Despite these relationships, we demonstrate that ants with ordinary *B. floridanus* levels exhibit higher mortality after exposure to the fungal entomopathogen *M. brunneum*, as compared to ants with reduced *B. floridanus* levels. Their more melanised cuticle thus did not thwart the conidiospores from penetrating, nor did internal immune responses slow the progression of the fungal infection [40,41,71]. In fact, the drastic increase in mortality by day four suggests accelerated fungal infection in ants with reduced *B. floridanus* levels. Although *C. floridanus* maintains normal systemic immune functions despite the symbiont [32], if *B. floridanus* is found outside of bacteriocytes, the ants elicit an immune response comparable to that of pathogenic bacteria [34]. During infection, *M. brunneum* can suppress the host immune system with secondary metabolites [42] and physically break host cells and tissues with its invading hyphae, which may allow *B. floridanus* to proliferate and escape bacteriocytes. This would require the host to elicit a multifaceted immune response involving both antifungal and antibacterial pathways of the innate immune system, analogous to a super-infection in insects where two distinct pathogens infect the host, hastening and increasing mortality [72,73,74,75]. Escaped *B. floridanus* in the haemocoel could thereby give the invading *M. brunneum* the upper hand in the battle against the host immune system. Histological analysis documenting the progression of the infection within the ant haemocoel linked to immune gene expression analysis is required to understand the mechanistic basis of this phenomenon. While the ants’ reduced resistance to a generalist fungal pathogen evinces a severe cost of hosting the primary endosymbiont, the generality of this phenomenon should be followed up by testing a wider range of generalist and specialist pathogens, including bacteria and viruses.

*Camponotus floridanus* nests in decomposing logs or in the ground beneath natural or manmade objects [67] and forages in the vegetation. They are thus likely to encounter a range of insect pathogens found in the environment, including conidiospores of *Metarhizium* fungi (see [76]). Additionally, their social lifestyle, which implies close and frequent contact with related colony members and unrelated myrmecophiles [36], exaggerates the risk of exposure to such pathogens and their spread within the colony [39,77]. Ants in general offset this enhanced risk of transmission with behavioural, physiological, and organisational defenses known as social immunity [38,39]. Self- and allogrooming, whereby individuals clean the cuticle of self and nestmates, are crucial behavioural measures in preventing individual infections, through limiting the number of, for example, fungal conidiospores that can firmly attach to, germinate on, and breach through the ant cuticle, to thereafter spread and proliferate as blastospores within the body cavity [42,78]. The additional positive effect of allogrooming is evidenced by an increased survival of *Metarhizium*-exposed ant workers kept in groups as compared to alone [79,80]. Along with other social behaviours, allogrooming may also mediate the spread of conidiospores among individuals within the colony, although mostly resulting in nonlethal infections [81]. However, in *C. floridanus* colonies, the cost associated with enhanced pathogen transmission is likely exacerbated, in that the increased susceptibility of all colony members means that the range for when a pathogen dose becomes lethal is lowered. This might have led to adaptive behavioural changes upon pathogen exposure that compensate their individual susceptibility, such as higher levels of both self- and allogrooming behaviours, as exhibited by *Camponotus sericeiventris* [76], or reduced social interactions, as displayed by *Camponotus aethiops* [82]. The risk of encountering pathogens becomes higher as ants move to activities outside the nest, which coincides with a reduction in *B. floridanus* levels [28]. Along with lessened nutritional requirements, the cost of an increased pathogen susceptibility may thus have selected for reduced endosymbiont levels in older workers, and also prompted changes in *Camponotus* social immunity in order to reduce the risk of individual infection and transmission within the colony. 

## 5. Conclusions

The primary endosymbiont *Blochmannia* provides essential nutritional supplementation to its host, *Camponotus*, which in the case of *C. floridanus*, facilitates growth and cuticle maturation in individuals and may contribute to colony polymorphism, a trait that is key to the success of the genus. The symbiont also imposes a critical trade-off in that it increases the ants’ susceptibility to fungal infection and thus secondarily raises the cost of pathogen transmission within the colony. The extensive coevolutionary relationship between the mutualistic partners and ubiquity of the symbiont throughout the genus [18,19,20,21,22] implies that the bacteria’s benefits to the ants’ life history, ecology, and evolution, outweigh the costs to their individual and social immunity. As highlighted throughout the discussion, further studies are required to understand the mechanistic underpinnings and evolutionary processes resulting in the observed trade-off.

## Supplementary Materials

The following are available online at http://www.mdpi.com/2075-4450/9/2/58/s1: Supplemental Methods and Results 1: Preliminary data set on cuticle melanisation: Figure S1: Cuticular melanisation of ants with reduced and ordinary symbiont levels; Supplemental Methods and Results 2: Symbiont reduction in callow ants and age-dependent melanisation; Figure S2: Head width and melanisation of callow ants; Figure S3: Age-dependent melanisation in ants with ordinary symbiont levels; Table S1: Benjamini–Hochberg-adjusted *p*-values Table S2; Colony census conducted four (Census 1) and seven months (Census 2) into the experimental diet period.

## Data Accessibility

The dataset is available from the Dryad repository; doi:10.5061/dryad.2pb6901.
