# Supplementary material for: Camponotusfloridanus Ants Incur a Trade-Off between Phenotypic Development and Pathogen Susceptibility from Their Mutualistic Endosymbiont Blochmannia
Source: Insects. 2018 Jun 1;9(2):58. doi: 10.3390/insects9020058 (PMC6023366; doi:10.3390/insects9020058)
Supplement: Supplementary file 1 [file insects-09-00058-s001.pdf]

*insects*

MDPI

*Communication*

# Endosymbiont *Blochmannia floridanus* mediates trade-off between development and disease defence in its carpenter ant hosts

**Veronica M. Sinotte [1,2] *, Samantha N. Freedman [1,3], Line V. Ugelvig [2] *, Marc A. Seid [1]**

**Supplemental Methods and Results**

## 1. Preliminary data set on cuticle melanisation

An initial experiment was completed to assess the impact of *Blochmannia floridanus* on the cuticular integrity of its host *Camponotus floridanus,* given that the symbiont is most abundant during stages of cuticle formation and development. We provided the ants with an antibiotic diet to reduce *B. floridanus*, and at the end of the experimental period we observed reduced melanisation in ants with depleted symbiont levels.

### *1.1. Ant culture*

Queenright colonies of *C. floridanus* were collected from Gainesville, Florida (29.6520° N, 82.3250° W). The colonies were housed in Fluon-lined containers, equipped with a cotton-plugged test tube of water and kept in the laboratory at 25°C with a 12-hour light/dark cycle. Four large colonies each containing >100 workers were selected for the experiment: two colonies were assigned an ordinary diet of *Tenibrio molitor* larvae and honey water (50% deionized water and 50% honey), and the remaining two colonies were assigned an antibiotic diet of *T. molitor* larvae and honey water with the antibiotic rifampicin (49.5% deionized water, 49.5% honey, 1% rifampicin). The ants were fed three times a week and maintained on the respective experimental diets for 6 months before melanisation was measured.

### *1.2. Assessment and analysis of cuticular melanisation*

Thirty randomly selected ants from each colony were used to assess cuticular melanisation of the thorax and gaster. Ants were photographed under a stereo microscope and the photos analyzed in ImageJ according to the protocol described in the main text. A general linear mixed model was used to determine the fixed effect of *diet* and *morphological location* on the log-transformed melanisation level, specifying *colony* as a random effect. The `lmer` function and packages `lme4` and `lmerTest` were used to create the model, and backwards model reduction was conducted ($p$>0.05) to obtain the minimum adequate model. Post hoc pairwise comparisons of *diet* and *morphological location* were conducted using least-squared means and Tukey $p$-value adjustment, employing the `lsmeans` package. The analysis was completed in R using R-Studio editor.

### *1.3. Results*

The interaction between *diet* and *morphological location* had a significant effect on ant melanisation (Figure S1; LMM; overall LR $\chi^2$= 18.116, *df*= 1, $p$<0.001). Ants receiving the antibiotic diet had a significantly lighter thorax and gaster compared to the respective areas of ants on the ordinary diet (post host comparisons, $p$=0.001, $p$<0.001 respectively). Due the similar effect of diet at both morphological locations we only measured the melanisation of the thorax in the main experiment.

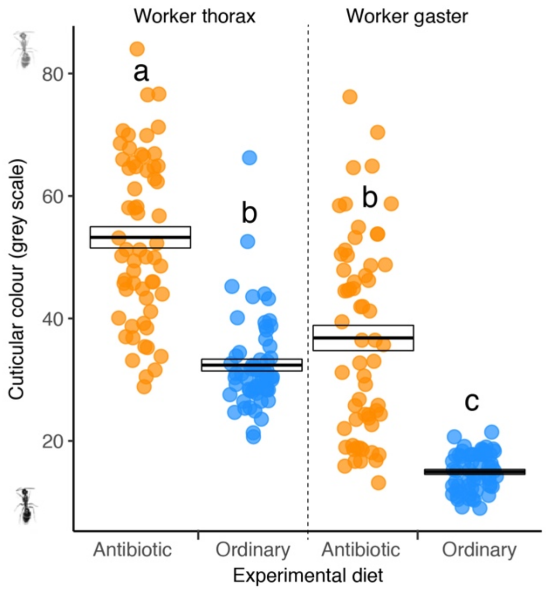


**Figure S1.** Cuticular melanisation of ants with reduced and ordinary symbiont levels. The colour of the thorax and gaster (displayed on a grey scale) of ants on the antibiotic diet (orange dots) compared to those on the ordinary diet (blue dots). Overlaying black boxes indicate the mean ± SE; letters specify significant post hoc groups [$\alpha = 0.05$].

## 2. Symbiont reduction in callow ants and age-dependent melanisation

We tested if the reduction of *B. floridanus* had an effect on the head width and melanisation of callow minor workers (10 days post-eclosion) and found that while head width decreased, melanisation was not significantly different. Second, we confirmed that cuticular melanisation is age-dependent in *C. floridanus*, with complete melanisation occurring between days 10 and 30 post-eclosion.

### *2.1. Ant culture*

In addition to the two age groups described in the main text, we assessed the head width and cuticular melanisation of a third age group of minor workers from the same colonies (note: one antibiotic-treated colony was substituted due to low fecundity and thus insufficient sample sizes). This group comprised minor workers developing from larvae to adult on the experimental diets sampled 10 days post-eclosion, at which point they were still callow workers.

### *2.2. Assessment and analysis of cuticular melanisation and head width*

Callow workers were randomly selected from each colony (immature-treated: $n_{antibiotic}=9$, $n_{ordinary}=9$) and the cuticular melanisation and head width were assessed according to the protocols described in the main text. General linear mixed models were used to determine the fixed effect of *diet* on head width and melanisation level, specifying *colony* as a random effect. A third model was conducted to determine the fixed effect of *age* on the melanisation of ants on the ordinary diet, with *colony* as a random effect. The `lmer` function and packages `lme4` and `lmerTest` were used to create the models, and backwards model reduction was conducted ($p>0.05$) to obtain minimum adequate models. Due to re-analysis of the data, *p*-values of the overall models were adjusted according to the Benjamini-Hochberg procedure. In all three cases, *p*-value adjustment did not alter the significance of the overall models (Table S1). A post hoc pairwise comparison of *age* was conducted using least-squared means and Tukey *p*-value adjustment, employing the `lsmeans` package. All analysis was completed in R using R-Studio editor.

### *2.3. Results*

The reduction of *B. floridanus* also is associated with smaller head width in callow minor workers (Figure S2A; LMM: overall LR $\chi^2$= 13.594, *df*= 1, *p*<0.001). However, symbiont reduction did not have a significant effect on their melanisation (Figure S2B; LMM: overall LR $\chi^2$= 1.429, *df*= 1, *p*=0.232), which may be explained by age-dependent cuticular melanisation (Figure S3; LMM: overall LR $\chi^2$= 38.111, *df*= 1, *p*<0.001), which completes between 10 and 30 days post-eclosion (post hoc comparison, *p*<0.001).

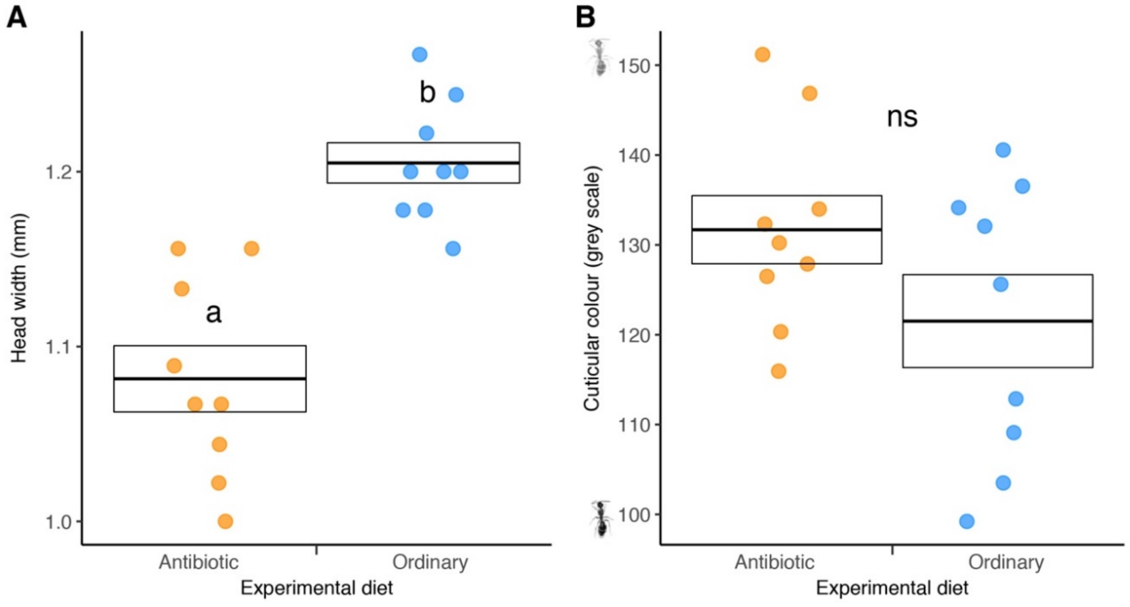


**Figure S2.** Head width and melanisation in callow ants. (**A**) Head widths and (**B**) cuticular melanisation in callow minors developing from egg to adult on the antibiotic diet (orange dots) compared to those on the ordinary diet (blue dots). Overlaying boxes indicate the mean ± SE; letters specify significant differences [$\alpha$ = 0.05].

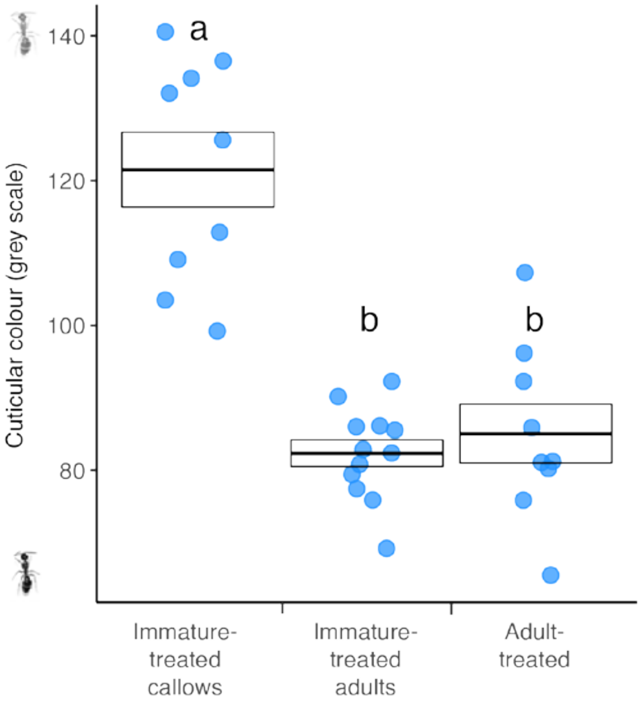


**Figure S3.** Age-dependent melanisation in ants with ordinary symbiont levels. The cuticular colour (displayed on grey scale) of ants across ages: 10, days (immature-treaded callows), 30 days (immature-treated adults), and several months (adult-treated) post-eclosion. Overlaying boxes indicate the mean ± SE; letters specify significant post hoc groups [$\alpha$ = 0.05].

**Table S1.** Benjamini-Hochberg adjustment of *p*-values for overall models displayed in Figures S2B and S3, due to re-analysis of data, are listed below.

| Analysis | Raw *p*-value | Adjusted *p*-value |
|---|---|---|
| Figure S2B | 2.319e-01 | 2.319e-01 |
| Figure S3 | 5.300e-09 | 4.268e-05 |

## 3. Colony growth and worker polymorphism

We tested if the reduction of *B. floridanus* had an effect on colony growth and worker polymorphism.

### *3.1. Assessment of colony size and worker poymorphism*

A census was obtained four months into the experimental diet period, the time required for an effect of the antibiotic treatment. Another census was completed across colonies after seven months of receiving the respective experimental diets, with one of the antibiotic-diet colonies exempt due to an ant escape during the experimental period. The overall number of workers removed for the measurement of head width and melanisation were added to the total number of minors in the census. These two census, allowed us to follow how colony size changed due to the different diets. At the second census, the number of major and minor workers were also obtained, enabling an estimate of worker polymorphism.

### *3.1. Results*

Colonies from the respective diets were of approximately similar size four months into the treatment (Table S2; average colony sizes: ordinary diet: 69, antibiotic diet: 65). In contrast, seven months into the experimental diet period, colonies on the ordinary diet had on average more than doubled in size, whereas colonies on the antibiotic diet had stagnated (average colony sizes: ordinary diet: 189, antibiotic diet: 60).

**Table S2.** Colony census obtained four (Census 1) and seven months (Census 2) into the experimental diet period. In Census 2, the number of majors, minors, and minors removed for head width and melanisation measures, were recorded (shown in italics).

| | **Colonies on ordinary diet** | | | | | | **Colonies on antibiotic diet** | | | | | |
|---|---|---|---|---|---|---|---|---|---|---|---|---|
| | **1C** | **2C** | **3C** | **4C** | **5C** | **6C** | **1A** | **2A** | **3A** | **4A** | **5A** | **6A** |
| **Census 1** | 111 | 83 | 56 | 71 | 45 | 49 | 68 | 60 | 62 | 100 | NA | 35 |
| **Census 2** | 187 | 130 | 187 | 237 | 208 | 183 | 76 | 46 | NA | 72 | 71 | 36 |
| *Majors* | *11* | *13* | *10* | *18* | *15* | *10* | *3* | *1* | *NA* | *1* | *2* | *0* |
| *Minors* | *167* | *117* | *168* | *207* | *193* | *173* | *70* | *45* | *NA* | *59* | *57* | *36* |
| *Removed* | *9* | *NA* | *9* | *12* | *NA* | *NA* | *3** | *NA* | *9** | *12* | *12* | *NA* |

© 2018 by the authors. Submitted for possible open access publication under the terms and conditions of the Creative Commons Attribution (CC BY) license (http://creativecommons.org/licenses/by/4.0/).
